# Data from wearable wireless instrumentation device taken during simulated CPR with and without intentional error toward a quality feedback device

**DOI:** 10.1016/j.dib.2019.104400

**Published:** 2019-09-11

**Authors:** Adam Pantanowitz, Sarah R. Ward, Bronwyn C. Scott, David M. Rubin

**Affiliations:** Biomedical Engineering Research Group, School of Electrical & Information Engineering, University of the Witwatersrand, Johannesburg, Private Bag 3, 2050, Johannesburg, South Africa

**Keywords:** Cardiopulmonary resuscitation (CPR), Quality, Dual-quaternions, Electromyogram (EMG), Inertial measurement unit (IMU), MYO, Machine learning

## Abstract

Data of Cardiopulmonary Resuscitation performed on a mannequin was collected via wearable instrumentation (using the MYO device). The data were collected for both “good” CPR and for performance of CPR with common errors introduced intentionally for this study. The data are labelled according to the error, and contain a variety of derived measurements. Data collected were used toward “Development of a novel cardiopulmonary resuscitation measurement tool using real-time feedback from wearable wireless instrumentation' (Ward et al., 2019) in which full context is available'. The data are available at Mendeley Data, doi:10.17632/pvjghfjmy4.1 (Ward et al., 2019).

**Table:** Specifications Table

Subject area	*Emergency Medicine*
More specific subject area	*Medical Engineering for Cardiopulmonary Resuscitation*
Type of data	*CSV file*
How data was acquired	*The first generation MYO was worn during the performance of CPR*
Data format	*Filtered and derived*
Experimental factors	*EMG values measured were normalised, orientation fields were derived using mathematical formulae such as quaternions*
Experimental features	*CPR was performed on a mannequin while wearing a MYO to measure acceleration and electromyogram (EMG). CPR was either performed as correctly as possible, or with intentional error, drawn from common errors. Measurements were labelled accordingly depending on the CPR.*
Data source location	*University of the Witwatersrand, Johannesburg, South Africa*
Data accessibility	*Available in a public repository at**https://doi.org/10.17632/pvjghfjmy4.1*
Related research article	Sarah R Ward, Bronwyn C Scott, David M Rubin, Adam Pantanowitz, “Development of a novel cardiopulmonary resuscitation measurement tool using real-time feedback from wearable wireless instrumentation”, Resuscitation, *in press*.

**Table:** 

**Value of the data** • Data allow for CPR motion measurements to be evaluated; • Data allow for CPR compression movement data to be evaluated for performance of quality CPR; • Data allow for CPR compression movement data to be evaluated for performance of poor quality CPR; • Data allow for Machine Learning Algorithm to be trained to determine quality & poor quality CPR, or for use in other CPR analysis; and • Data contain a number of repeated measurements of CPR.

## 1 Data

The data are presented in two Comma-Separated-Value (CSV) files [2]. Fields are described in Table 1, with labels corresponding as per the data file. Table 2 outlines a brief description of the hand/body positions used for the various classes of CPR (“Good” CPR and for the introduction of errors in CPR) as per [1]. The remaining fields are associated with Electromyogram (EMG) data measured as Root Mean Square (RMS) values, accelerometer data, and zero-crossing data as described in Table 1.

**Table 1:** Data Description Table for *combinedData.data*.

Column (field)	Description	Data Label
0	CPR Quality Label as per Table 2	*label*
1–8	RMS data from each of the 8 EMG channels	*rms1 to rms8*
9–15	the ratio of the first EMG channel to each of the other channels (except itself)	*r1-2 to r1-8*
16–21	the ratio of the second EMG channel to each of the other channels (except itself and channel 1)	*r2-3 to r2-8*
22–26	the ratio of the third EMG channel to each of the other channels (except itself and channel 1 and 2)	*r3-4 to r3-8*
27–30	the ratio of the fourth EMG channel to each of the other channels (as above)	*r4-5 to r5-8*
31–33	the ratio of the fifth EMG channel to each of the other channels (as above)	*r5-6 to r6-8*
34–35	the ratio of the sixth EMG channel to each of the other channels (as above)	*r6-7 and r6-8*
36	the ratio of the seventh EMG channel to the eighth	*r7-8*
37–44	the zero crossing rate of each channel	*zerocrossing1 to zerocrossing8*
45–48	the average orientation data in quaternion form	*orientation1 to orientation3*.

**Table 2:** CPR quality labels with intentional Errors and associated hand positions, as depicted visually in [1].

Label in column (field) 0	Description
Good	Good (focusses on hand position and body position for descriptive purposes in [1])
Relaxed	Shallow compressions due to relaxed hands (fingers curled, intentional incorrect hand position)
Sideways	Hands positioned too low on the chest (intentional incorrect position)
Good	Good (focusses on hand position and body position for descriptive purposes [1])
Forward	Body position is too forward over the patient (intentional incorrect practitioner body position)
Far	Body position is too far from the patient (intentional incorrect practitioner body position)

*combinedData.txt* is raw measured data, and includes a label of “Junk”, which is experimental throwaway data. A total of 4 637 instances were measured.

*combinedData.data* is post-processed to remove “Junk” labels, which the authors determined not to be useful, and shuffles raw data. This set is the set used as training data to train the models as per [1]. A total of 4 252 instances remain after filtering out the “Junk” class.

Processed output data (training data for machine learning) is in *combinedData.data*.

Field 0 is associated with CPR quality (the outcome label), and so this is described more extensively in Table 2.

## 2 Experimental design, materials, and methods

The correct position for performance of CPR is with the heel of the hand on the sternum in the middle of the line connecting the nipples [3]. Fingers should not rest on the patient, and arms should be perpendicular to the patient's sternum [3]. The correct position and most common positional mistakes are presented with photographs in [1]. Being too far from the patient causes the CPR practitioner's arms to be at less than ninety degrees to the patient's sternum, thus the force of compressions is directed at an angle instead of vertically down towards the patient's heart. Being too close to the patient has a similar effect with the difference being that the angle of the practitioner's arm with the patient's sternum is larger than ninety degrees. Resting fingers on the patient's chest distributes the force over a larger area thus transferring less force (and hence compression) to the heart. Placing hands too low on the sternum risks unnecessarily breaking ribs and reduces the force transferred to the heart as the hands are below the location of the centre of the heart.

These correct and incorrect hand and body positions for CPR practice are intentionally performed on a CPR mannequin and captured in the data set, as per Table 1, Table 2, and depicted visually in [1]. The Myo was used to collect data by performing good CPR as well as CPR with intentional error, as described and labelled.

The Myo SDK combines the acceleration, gyroscope and magnetometer data into orientation data in the form of a quaternion ($\bar{q}$), consisting of three complex vectors and a scalar value. The use of quaternions reduces accelerometer and gyroscope error by comparing them to an absolute frame of reference provided by the magnetometer.

The roll (ϕ), pitch (θ) and yaw (ψ) are calculated, with roll and pitch giving information about the sideways position and the forwards/backwards positions of the arms respectively.

The orientation provides a further three features for machine learning and is significant as it aids in determining the angle of the arm (particularly for hands too low on the sternum, too far away, and too close.

The following information is based on [4], [5].

$$\bar{q}=\left[\begin{array}{c} a\\ b\\ c\\ d \end{array}\right]=\left[\begin{array}{c} s_{x}sin\left(\frac{\gamma }{2}\right)\\ s_{y}sin\left(\frac{\gamma }{2}\right)\\ s_{z}sin\left(\frac{\gamma }{2}\right)\\ cos\left(\frac{\gamma }{2}\right) \end{array}\right]$$

$$\begin{array}{rr} where\;s & =vector\;distance,\\ \gamma & =vector\;rotation \end{array}$$

$$\phi =tan^{-1}(\frac{2(ad+bc}{-a^{2}-b^{2}-c^{2}+d^{2}})$$

$$\theta =sin^{-1}\left(2\left(ac-bd\right)\right)$$

$$\psi =tan^{-1}(\frac{2(cd+ab}{a^{2}-b^{2}-c^{2}+d^{2}})$$

### 2.1 Rate

A Fourier Transform converts the time varying acceleration data into the frequency domain. The Discrete Fourier Transform (DFT) from OpenCV [6] is used since this is used for machine learning, reducing memory requirements. The OpenCV DFT for one dimensional arrays is utilised [6]. The magnitude is calculated from the resulting array, giving a frequency spectrum containing the rate of compressions in the expected 1.6–2 Hz range [7]. This signal is low pass filtered with a cut-off frequency of 3Hz to eliminate harmonics and determine the fundamental component.

$$F_{jk}^{N}=e^{\frac{-2*pi*ijk}{N}}$$

$$\bar{q}=\left[\begin{array}{c} a_{0}\\ b_{0}\\ c_{0}\\ d_{0} \end{array}\right]=\frac{1}{2}\left[\begin{array}{cccc} z & 0 & -x & y\\ 0 & -z & y & x\\ -y & x & 0 & z\\ -x & -y & -z & 0 \end{array}\right]\left[\begin{array}{c} a\\ b\\ c\\ d \end{array}\right]$$

### 2.2 Depth

The current quaternion is compared with the previous quaternion in order to find the incremental distances $s_{x}$, $s_{y}$and $s_{z}$. The absolute values of these incremental distances are added and averaged for the given window and divided by two in order to account for both the rising and falling directions of the compressions. The distance obtained is then multiplied by a constant in order to scale distances to centimetres.

$$s_{x}=2\left(-ac_{0}+bd_{0}+ca_{0}-db_{0}\right)$$

$$s_{y}=2\left(ad_{0}+bc_{0}-cb_{0}-da_{0}\right)$$

$$s_{z}=2\left(ab_{0}-ba_{0}+cd_{0}-dc_{0}\right)$$
